# Effect of temperature on microbial communities in bentonite for use in engineered barrier systems

**DOI:** 10.1128/msphere.00313-25

**Published:** 2025-08-08

**Authors:** Rachel C. Beaver, Cailyn M. Perry, Chang Seok Kim, Josh D. Neufeld

**Affiliations:** 1 Department of Biology, University of Waterloo 8430, Waterloo, Ontario, Canada; 2 Nuclear Waste Management Organization (NWMO) 278230, Toronto, Ontario, Canada; Third Institute of Oceanography Ministry of Natural Resources, Xiamen, China

**Keywords:** bentonite, sulfate-reducing bacteria, temperature, nuclear waste storage, bacteria, deep geological repository

## Abstract

Bentonite is an important component of deep geological repository (DGR) designs, where it will serve as a buffer between used fuel containers (UFCs) and subsurface rock walls of the repository. The potential for microbial activity in bentonite is being studied to understand the influence of microbial metabolisms (e.g., sulfate reduction by sulfate-reducing bacteria) on the long-term safety of the DGRs (e.g., through contributions to microbiologically influenced corrosion). Most studies of microorganisms in bentonite involved culturing microorganisms at 30°C or below, even though the placement room in a DGR is expected to experience elevated temperatures for up to one thousand years after used fuel placement. The purpose of this study was to test the abundance and community composition of microorganisms in as-received and hydrated bentonite at a range of DGR-relevant temperatures (15–105°C) using a combination of cultivation and DNA-based techniques. In certain clays tested, aerobic heterotrophs, anaerobic heterotrophs, and sulfate-reducing bacteria were culturable at 15, 30, 45, and 60°C from both the as-received and hydrated bentonite, demonstrating that the bentonite microbial community includes representatives capable of growth at a range of DGR-relevant temperatures. Although cultivation results showed no significant increase in the abundance of culturable microorganisms from as-received bentonite to hydrated bentonite at temperatures greater than 45°C, sequencing results for two bentonite samples hydrated and incubated at 60°C revealed that 16S rRNA gene profiles were dominated (>99%) by sequences associated with the putative thermophilic family Thermoactinomycetaceae, which was not detectable in the as-received bentonite starting material. Not only does this suggest that as-received bentonite harbours viable thermophiles, but it also highlights the importance of a multifaceted (e.g., cultivation coupled to DNA sequencing) approach to study microbial communities of bentonite.

**IMPORTANCE:**

Predicting the abundances and types of microorganisms that may be active within a deep geological repository is critical to ensure that DGR design specifications minimize or prevent microbially mediated deterioration of DGR components. To date, research in this area has focused on the effect of bentonite dry density and the associated swelling pressure on suppression of microbial growth, but most of these experiments have been conducted at relatively low temperatures (e.g., 30°C). Studying the microbiology of bentonite exposed to elevated temperatures is critical given that a DGR is expected to experience high temperatures for up to one thousand years.

## INTRODUCTION

Bentonite clay is an important component of proposed deep geological repository (DGR) designs for long-term storage of used nuclear fuel, where it will serve as a buffer between used fuel containers (UFCs) and the host rock. In terms of microbiology, it is important to understand the response of bentonite-associated microbial communities to the conditions they are likely to experience in a DGR to better predict the long-term stability of such a repository. Studies on the microbiology of bentonite in the context of DGR design often focus on sulfate-reducing bacteria (SRB), which produce corrosive sulfide, because they commonly dominate occurrences of microbiologically influenced corrosion (MIC) in other anoxic environments (1). In a DGR, MIC could potentially compromise the integrity of metal UFCs, which house the used nuclear fuel. Despite focusing on SRB, microorganisms often live in communities with other microorganisms, where they may have various symbiotic relationships, such as with microorganisms that decompose complex organic matter, produce H_2_ that some SRB can use as an electron source, or oxidize sulfur to produce sulfate (2). As such, it is important to study microbial communities of bentonite rather than focusing only on specific populations. One important goal in designing DGRs, including the Canadian DGR, is to identify the minimum dry density of bentonite that is required to suppress microbial growth. As such, the major area of focus for bentonite microbiology studies has been the effect of dry density on microbial abundance and survival (3–10).

In addition to dry density, an important factor that has been less well studied in bentonite microbiology research is temperature. A DGR is expected to experience a wide range of microbiologically relevant temperatures. Before the construction of a DGR, the temperature in the subsurface of the Canadian host site, at repository depth, is approximately 12°C (11). Within a DGR, used nuclear fuel, which generates heat, will increase the temperature within the DGR over time (11). The DGR is being designed in such a way that the temperature within the system never reaches levels high enough to convert bentonite to non-swelling illite clay or cause electrochemical changes in the copper coating of the UFCs that could increase corrosion rates (12). Specifically, the Canadian DGR is being designed so that UFC surface temperatures do not exceed 100°C (12). The expected temperature that may be reached within different areas of a DGR depends on the type of rock at the host site. In the proposed Canadian DGR, models show a steady temperature increase in the area surrounding UFCs until a maximum of 84°C is reached ~45 years after emplacement, after which temperatures are expected to remain elevated (>70°C) for over 1,000 years before cooling over several thousand years. Overall, the expected temperature range within a DGR is anywhere between 12°C and 84°C, depending on the location within the DGR and the time after used fuel placement (11).

Very few studies have investigated microbial community composition and abundance in bentonite exposed to elevated temperatures. In bentonite from El Cortijo de Archidona (Spain), heterotrophs and SRB did not increase in abundance when bentonite was compacted to a dry density of 1.7 g/cm^3^ and incubated under anoxic conditions at 60°C for a year, but they did remain culturable in similar abundances before and after compaction and incubation (8). Even in bentonite first sterilized through a “Tyndallization” process (i.e., repeated heating and cooling) prior to compaction, microorganisms from the clay plugs were culturable, though in lower numbers than the starting material (8). This suggests that native bentonite microbial communities may not proliferate under harsh temperature and dry density conditions, but they can survive the conditions and grow once temperature and dry density are reduced. In another study exploring the effect of temperature on microbial communities within Bavarian bentonite in the absence of pressure, sulfate reduction was evident in slurries of Bavarian bentonite and Opalinus clay porewater solution incubated at 30°C, but not in parallel 60°C incubations (13). However, a shift in microbial community composition was observed through DNA sequencing of the 60°C slurry. The 16S rRNA gene profiles of these high-temperature microcosms were dominated by sequences associated with the thermophilic species *Caldinitratiruptor microaerophilus* (13). Sequences associated with this same thermophilic genus, as well as *Brockia*, *Thermaerobacter*, and *Thermincola*, were also detected in Czech calcium-magnesium (BCV) bentonite suspensions heated to 60 or 70°C (14). In this study, DNA-based evidence suggested that microbial survival was limited by a temperature of 90°C, though other studies on the same type of bentonite demonstrated low levels of culturable cells remaining after 6 months of incubation at 90°C (15) or multiple rounds of 121°C dry heat sterilization (16).

**Fig 1:**
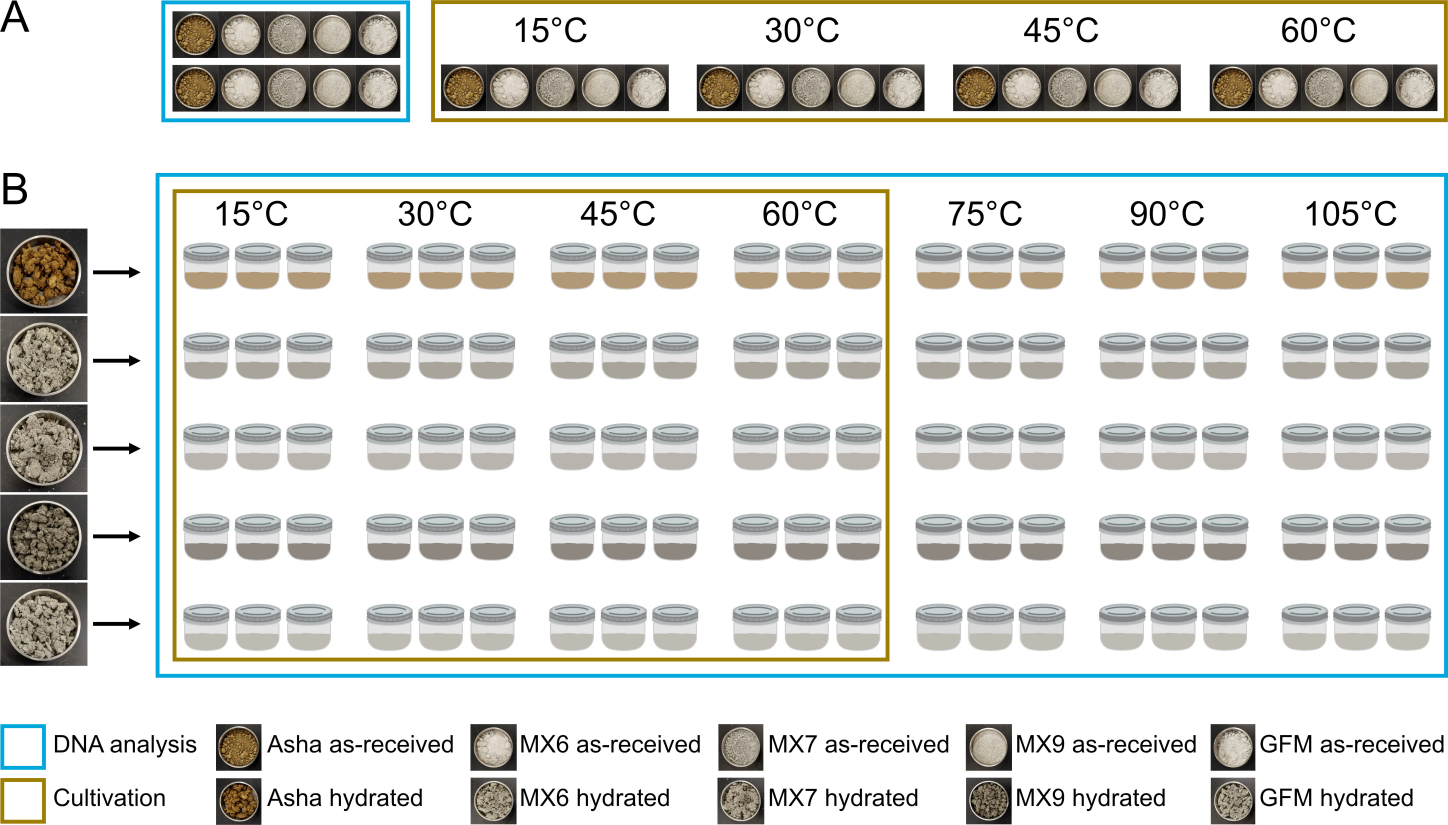
Schematic of experimental setup of as-received bentonite (**A**) and hydrated bentonite (**B**) DNA and cultivation-based analyses for five bentonite types.

Our goal was to investigate the effect of temperature on microbial community composition in as-received bentonite (i.e., relatively dry clay, “as-received” from the manufacturer) and bentonite hydrated to DGR-relevant water activities and moisture contents. Bentonite in the Canadian DGR is predicted to take up to 50 years to fully saturate, and bentonite moisture content and water activity will additionally be dictated by its dry density (17). As bentonite water activity and moisture content are expected to fluctuate in early repository phases, we chose to simulate a “worst case scenario” high water activity situation by hydrating as-received bentonite to a high water activity of 0.99. We examined five as-received bentonite samples, including Wyoming MX-80 bentonite and gapfill bentonite (i.e., granulated bentonite produced from Wyoming MX-80 through a low-temperature roll-compaction method), both of which are proposed for use in a Canadian DGR, as well as Asha bentonite, which has previously been observed to have higher abundances of culturable microorganisms than Wyoming MX-80 bentonite (18). For each, we studied the effect of a range of DGR-relevant temperatures (15, 30, 45, 60, 75, 90, and 105°C) on microbial community composition and abundance, with and without hydration (Fig. 1). Although a DGR is expected to be anoxic for most of its lifetime, we conducted this initial set of experiments under oxic conditions to simulate the early oxic repository phase, during which microbial growth may be elevated prior to oxygen depletion.

## RESULTS

### Mineral characterization of as-received bentonite

Of the five clays used in this study, three (MX6, MX7, and MX9) are different batches of Wyoming MX-80 bentonite. These three bentonite samples were composed of 77.3–88.4% montmorillonite, the primary mineral in bentonite (Table 1). Other minerals, such as plagioclase, feldspar, quartz, mica, calcite, and gypsum, were present at lower abundances, and cristobalite was additionally detected in MX6 and MX7, but not MX9 (Table 1). The fourth bentonite, gapfill material (GFM), was similar in mineral composition to MX6 and MX7, though it included clinoptilolite (6.7%; a type of zeolite that, like bentonite, has strong adsorption and ion exchange properties), which was not detected in any of the other bentonites (Table 1) (19). Finally, Asha bentonite, which was sourced from Kutch, India, was most similar to MX9 in abundance of montmorillonite, quartz, and plagioclase. Asha bentonite did not include feldspar or mica, which were detected in all other bentonite samples, but did include kaolinite, gypsum, and goethite, which were either not detected or detected at trace abundance in the remaining samples (Table 1).

**TABLE 1:** Mineral composition (%) of the five bentonite clays used^*a*^

Mineral	Montmorillonite	Quartz	Cristobalite	Plagioclase	K feldspar	Mica	Clinoptilolite	Kaolinite	Calcite	Gypsum	Goethite
Asha	84.4	1.4	ND	4.8	ND	ND	ND	3	3.1	1.4	1.9
MX6	77.3	3.9	1.4	9.5	4.1	2.2	ND	ND	1.6	Trace	ND
MX7	77.5	3.7	1.5	11.8	3.1	1.7	ND	ND	0.7	Trace	ND
MX9	88.4	1.3	ND	4.6	2.8	1.1	ND	ND	1.8	Trace	ND
GFM	73.7	1.6	2.7	10.4	2.9	2	6.7	ND	trace	Trace	ND

^*a*^ When the mineral was not detected, it is reported as “ND,” and when detected in amounts too low to quantify is reported as “trace”.

### Cultivation of bentonite from microcosms

**Fig 2:**
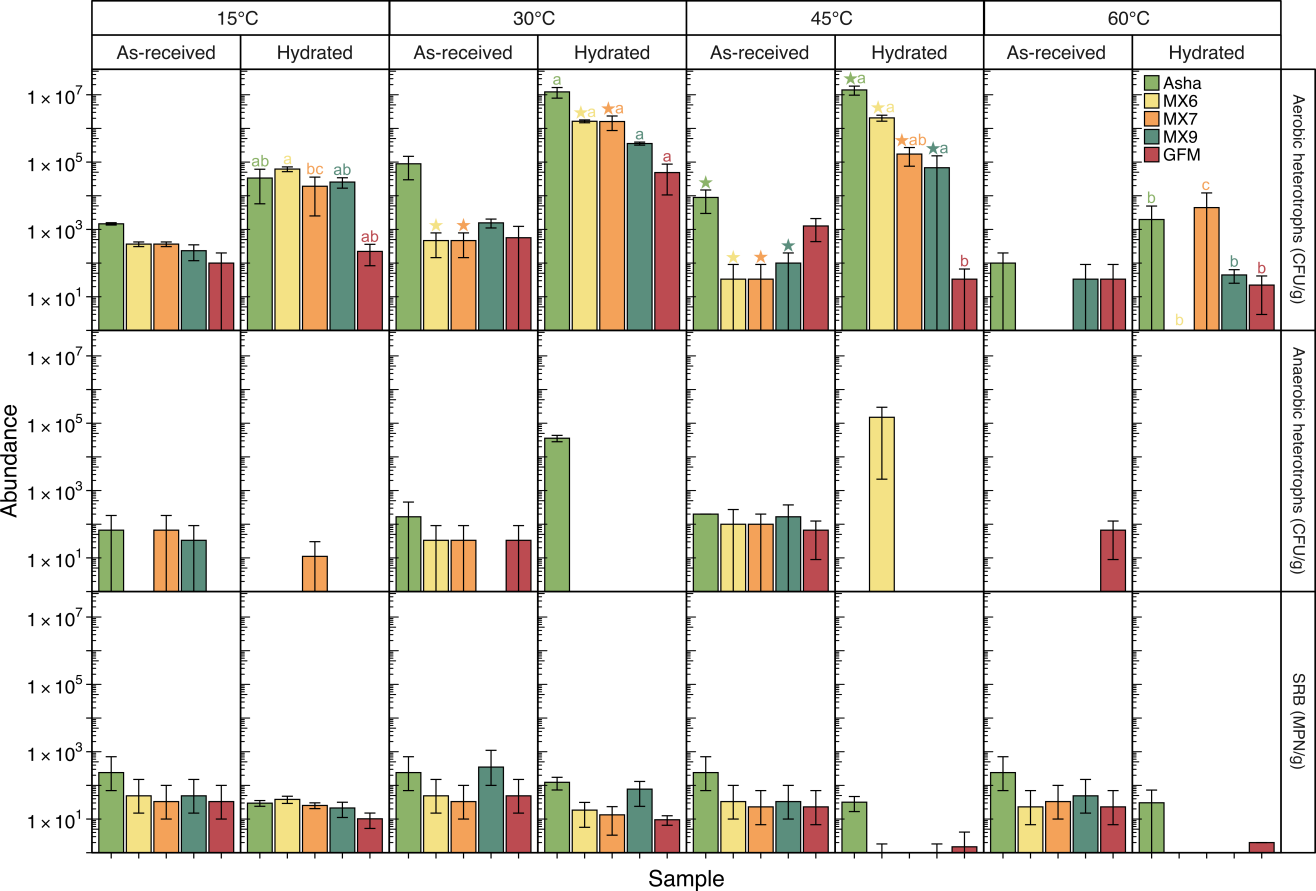
Bar plot showing the abundance of culturable aerobic heterotrophs, anaerobic heterotrophs, and sulfate-reducing bacteria (SRB) grown at 15, 30, 45, or 60°C from as-received or hydrated Asha, MX6, MX7, MX9, or GFM bentonite. Error bars represent standard deviation between technical triplicates (heterotrophs) or the 95% confidence intervals from the MPN table (SRB) for as-received clays, and the standard deviation between biological triplicates for hydrated clays. Stars in the aerobic heterotrophs panel indicate significant differences in CFU/g before and after hydration at the given temperature (Tukey’s HSD test, *P* < 0.05). Small letters in the aerobic heterotroph panel denote statistical groupings across temperature for each hydrated clay (Tukey’s HSD test, *P* < 0.05). No letters are included for dry clays, where temperature had no significant effect (Tukey’s HSD, *P* < 0.05).

Culturable aerobic heterotrophs were detected in each of the clays, dry or hydrated, when cultures were incubated at 15, 30, or 45°C (Fig. 2). Culturable aerobic heterotrophs grew at 60°C from as-received Asha, MX9, and GFM samples, and from all but MX6 when the clay was first hydrated and incubated at 60°C for a week (Fig. 2). At 30°C, there were more aerobic heterotrophs cultured from hydrated clay compared to as-received clay for MX6 and MX7, and the same was true for 45°C cultures for all clays other than GFM (Fig. 2 stars; Tukey’s HSD, *P* < 0.05). For aerobic heterotroph cultures grown at 15 or 60°C, the abundance before and after hydration was not significantly different for any bentonite type (Fig. 2; Tukey’s HSD, *P* < 0.05). For each hydrated clay, the highest abundance of culturable aerobic heterotrophs was either at 30 or 45°C (Fig. 2). The value of culturable aerobic heterotrophs at 30 and 45°C was statistically the same for all clays other than GFM, for which the abundance at 30°C was greater than that at 45°C, and was often also the same as the abundance at 15°C (Fig. 2 letters; Tukey’s HSD, *P* < 0.05). The abundance of culturable aerobic heterotrophs at 60°C for each hydrated clay was significantly lower than for the same hydrated clay incubated at 30°C. For all as-received clays, there was no statistical difference between the abundance of aerobic heterotrophs cultured at any of the four temperatures (Tukey’s HSD, *P* < 0.05). Several estimates of heterotroph abundance were associated with very few colonies: all counts of CFU/g < 2,000 are associated with average plate counts of <20 colonies/plate, and some abundance estimates are based on plates with as few as a single colony. These very low culture counts often resulted in perceived high variation (i.e., large error bars) among replicates, as has been observed in other low-biomass bentonite studies (4, 5, 10, 20–22), and should generally be interpreted cautiously.

Culturable anaerobic heterotrophs were detectable in fewer samples than for aerobic heterotrophs (Fig. 2). Anaerobic heterotrophs were cultured from all as-received bentonites at 45°C, from all but MX9 at 30°C, and from all but MX6 and GFM at 15°C (Fig. 2). At 60°C, the only as-received clay from which anaerobic heterotrophs were culturable was GFM (Fig. 2). For all tested conditions, the abundance of culturable anaerobic heterotrophs in as-received clay was low, with a maximum value of 127 CFU/g. Once hydrated and incubated for a week, most clays did not have culturable anaerobic heterotrophs regardless of whether they did initially in the as-received clay (Fig. 2). At each temperature below 60°C, one hydrated clay had culturable anaerobic heterotrophs, and no clay had culturable anaerobic heterotrophs at more than one tested temperature (Fig. 2).

SRB were cultured from as-received clay at all four tested temperatures for all bentonite types, at a maximum abundance of 144 MPN/g (Fig. 2). They were culturable from all hydrated clays incubated at 15 or 30°C, at similarly low abundances, but were often not detectable in clays hydrated and incubated at 45 or 60°C (Fig. 2). At 60°C, SRB were only culturable from Asha and GFM, and at 45°C from all but MX7; however, for MX6 and MX9, only one tube showing SRB growth was observed, each in only one of three biological replicate microcosms (Fig. 2).

### 16S rRNA gene analysis of as-received bentonite and associated cultures

Before proceeding with sequence analysis of bentonite or culture samples, 16S rRNA gene profiles of samples were compared to those of controls. In total, 106 negative controls, including 35 DNA extraction kit controls and 71 no template PCR controls, were included in the sequencing runs. Of the 106 negative controls, only 14 had greater than 100 reads after processing with Decontam (Fig. S1). There were no ASVs that were consistent across all controls. Instead, control profiles appeared to be stochastic, with each control represented by one or a few ASVs with low read counts (Fig. S1). Overall, analysis of controls did not suggest contaminant-dominated profiles for most samples, especially those with relatively high read count and consistency among replicates.

**Fig 3:**
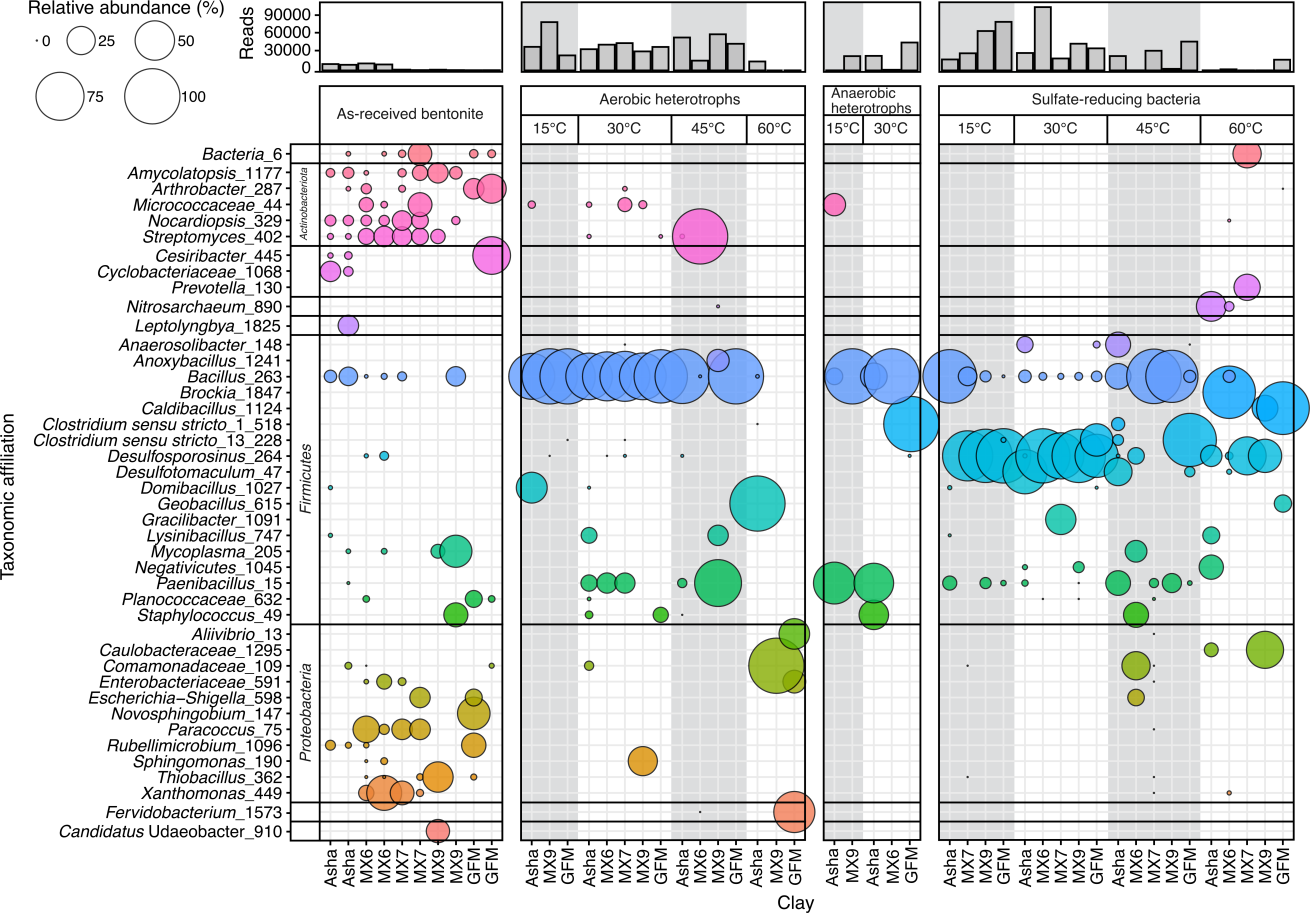
Bubble plot of 16S rRNA gene profiles of as-received bentonite samples and cultures set up from as-received bentonite samples and incubated at 15, 30, 45, or 60°C. Only genera with a minimum relative abundance of 5% in at least one sample are displayed. The bar plot displays the number of sequence reads obtained for each sample.

Although there were differences in 16S rRNA gene profiles among the five different as-received bentonite types, these profiles were generally dominated by sequences associated with phyla Actinobacteriota and Proteobacteria (Fig. 3). The most abundant genera among as-received clay samples were *Amycolaptosis*, *Arthrobacter*, *Nocardiopsis*, *Streptomyces*, *Bacillus*, *Paracoccus, Xanthomonas*, and family Micrococcaceae (Fig. 3).

Whereas as-received clays were dominated by sequences associated with phyla Actinobacteriota and Proteobacteria, most cultures from as-received clays were dominated by sequences associated with phylum Firmicutes, regardless of clay, culture type, or incubation temperature (Fig. 3). In aerobic heterotroph cultures incubated at 15 or 30°C, all 16S rRNA gene profiles were dominated (i.e., 68.5–100.0%) by sequences associated with *Bacillus*. At 30°C, sequences associated with *Paenibacillus* also had relatively high abundances in Asha, MX6, and MX7 profiles, and *Sphingomonas* and *Staphylococcus* in MX9 and GFM, respectively (Fig. 3). Aerobic heterotroph cultures incubated at 45°C were either dominated by sequences associated with *Bacillus* (Asha and GFM), *Streptomyces* (MX6), or *Paenibacillus* (MX9). The aerobic heterotroph cultures incubated at 60°C had no overlap of dominant genera for the three clays from which aerobic heterotrophs were culturable (Fig. 3). That said, biomass extracted from MX9 and GFM cultures incubated at 60°C had very low numbers of sequencing reads (i.e., 8 and 44, respectively).

Similar to aerobic heterotroph cultures, anaerobic heterotroph cultures incubated at both 15 and 30°C also often had relatively high abundances of sequences associated with *Bacillus* in all but the GFM 30°C cultures, which were instead dominated by sequences affiliated with *Clostridium sensu stricto 1* (Fig. 3). The genus *Paenibacillus* was also represented by relatively abundant reads in Asha cultures incubated at both 15 and 30°C (Fig. 3).

The dominant putative SRB represented in the 16S rRNA gene profiles of SRB cultures were associated with the genus *Desulfosporosinus*, although *Desulfotomaculum* sequences dominated Asha cultures incubated at 15 or 30°C and were additionally detected in lower relative abundance in cultures from GFM and MX6 incubated at 45 and 60°C, respectively (Fig. 3). Sequences associated with *Desulfosporosinus* and/or *Desulfotomaculum* were detected in all SRB cultures from as-received clay except for SRB cultures from Asha incubated at 15°C, from MX7 and MX9 incubated at 45°C, and from GFM incubated at 60°C, which were instead dominated by *Caldibacillus* (GFM at 60°C) or *Bacillus* (Asha, MX6, MX7, MX9; Fig. 3).

### 16S rRNA gene analysis of hydrated bentonite and associated cultures

**Fig 4:**
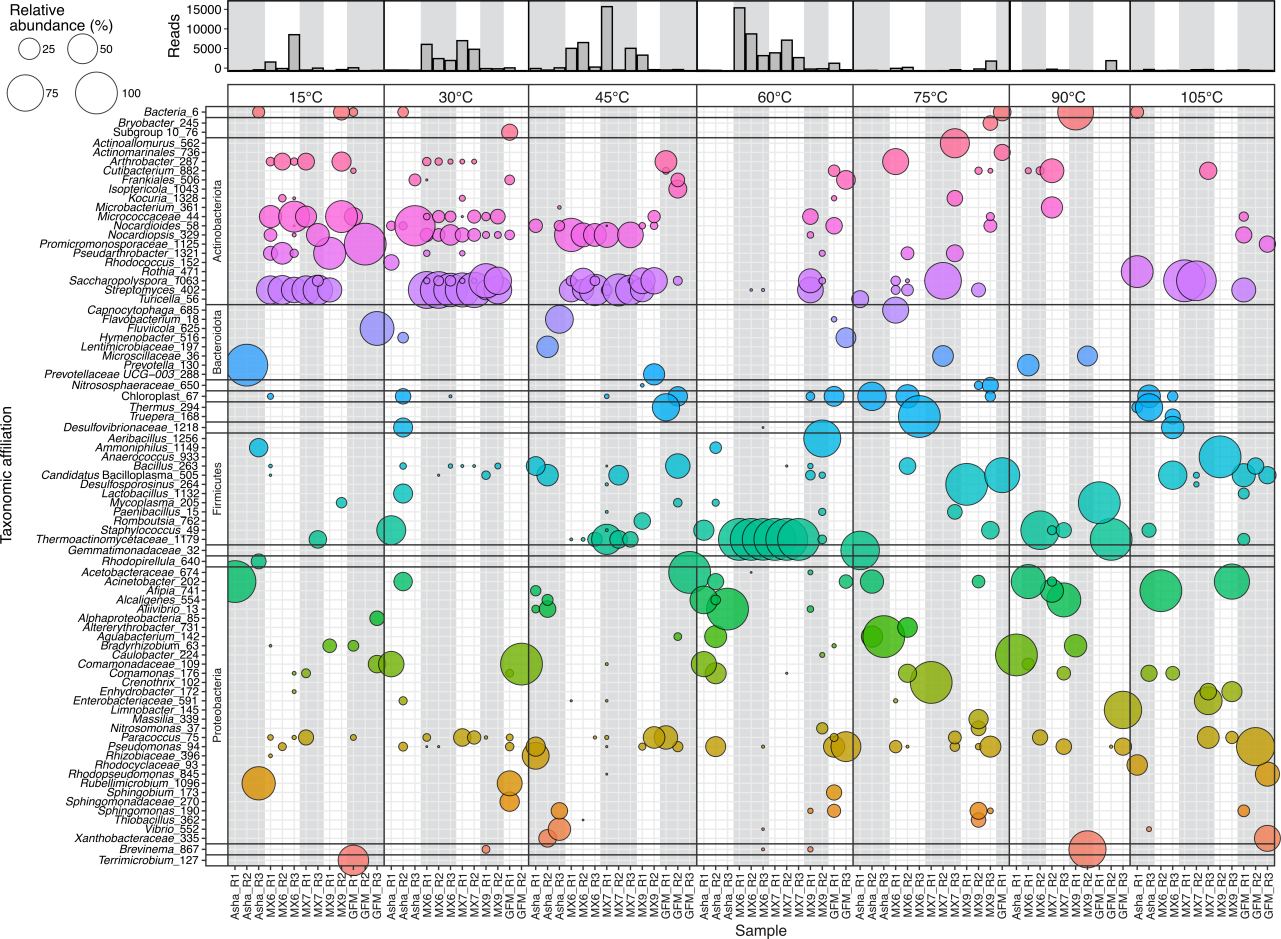
Bubble plot showing the 16S rRNA gene profiles of five bentonite samples (three biological replicates each) hydrated with Type 1 water and incubated for seven days at 15, 30, 45, 60, 75, 90, or 105°C. Only genera with a minimum relative abundance of 10% in at least one sample are shown. The bar plot displays the total number of sequence reads for each sample.

**Fig 5:**
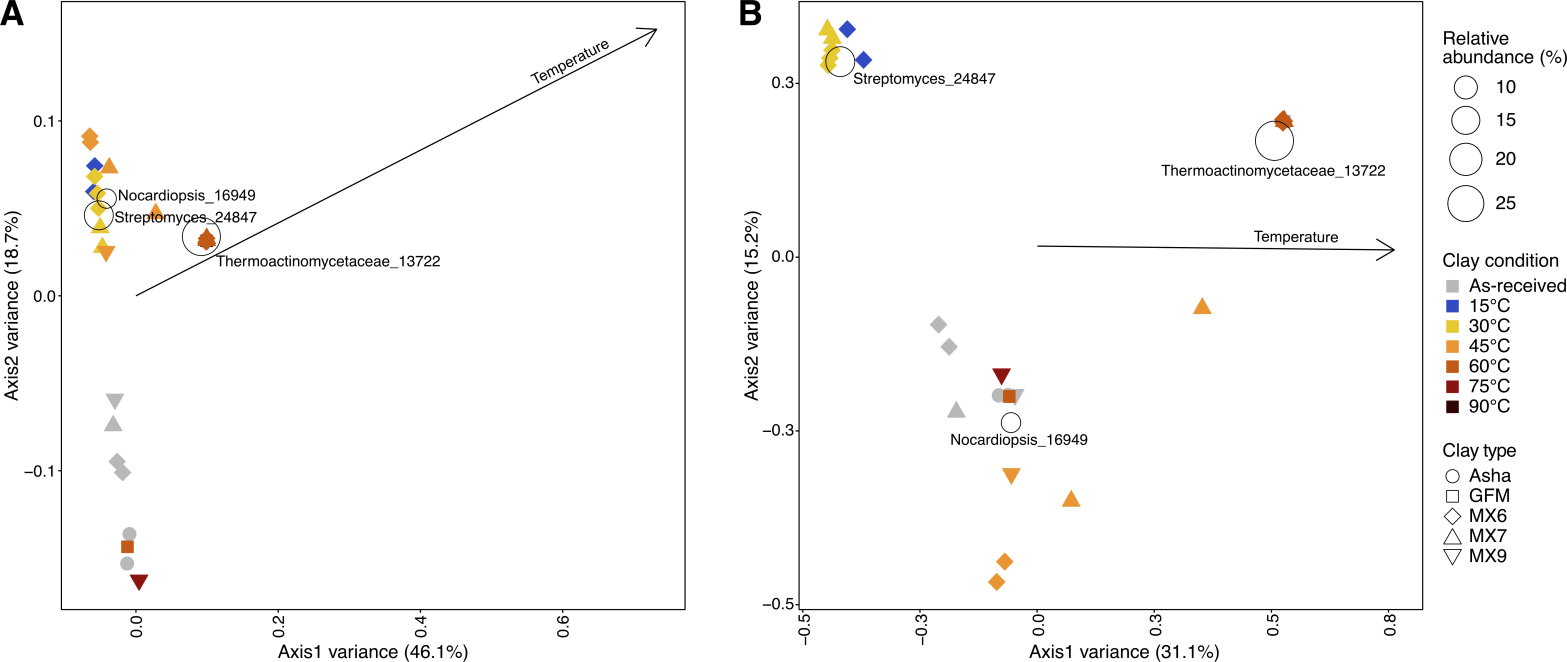
Triplots based on weighted UniFrac (**A**) and Bray–Curtis (**B**) distances between 16S rRNA gene profiles of as-received bentonite and hydrated bentonite incubated at 15, 30, 45, 60, 75, or 90°C. The vectors represents the correlation of temperature with the ordination (*R*^2^ = 0.54 and *P* = 1 × 10^−4^ for weighted UniFrac, *R*^2^ = 0.58 and *P* = 1 × 10^−4^ for Bray–Curtis). Taxa bubbles represent taxa that have a minimum relative abundance of 5% across the data set. The ASV table was normalized to 1,000 reads using scaling with ranked subsampling (SRS) prior to computing distances.

The 16S rRNA gene profiles of hydrated clay samples were often dominated by similar sequences when the bentonite was incubated at 15, 30, or 45°C (Fig. 4). The three Wyoming MX-80 bentonites (MX6, MX7, and MX9) had profiles dominated by sequences associated with *Streptomyces*. Sequences associated with Micrococcaceae were also relatively abundant when the bentonite was incubated at 15 or 30°C, and sequences associated with *Nocardiopsis* were abundant when bentonite was incubated at 30 and especially at 45°C (Fig. 4). Hydrated Asha and GFM replicates had relatively low sequence read abundances, likely contributing to replicate profiles with little overlap. In ordination space, all hydrated clays incubated at 15 or 30°C grouped together—both when weighted UniFrac (Fig. 5A) and Bray–Curtis (Fig. 5B) distances were used—and separately from the un-hydrated, as-received bentonite. The bentonite incubated at 45°C had 16S rRNA gene profiles that overlapped with 15 and 45°C bentonite samples, but also had comparatively higher relative abundances of sequences associated with *Nocardiopsis*, which was prevalent and relatively abundant in as-received bentonite samples (Fig. 4). In ordination space, these 45°C samples grouped with the 15 and 30°C samples when weighted UniFrac distances were used (Fig. 5A), but grouped more closely with starting material when Bray–Curtis distances were used (Fig. 5B).

**Fig 6:**
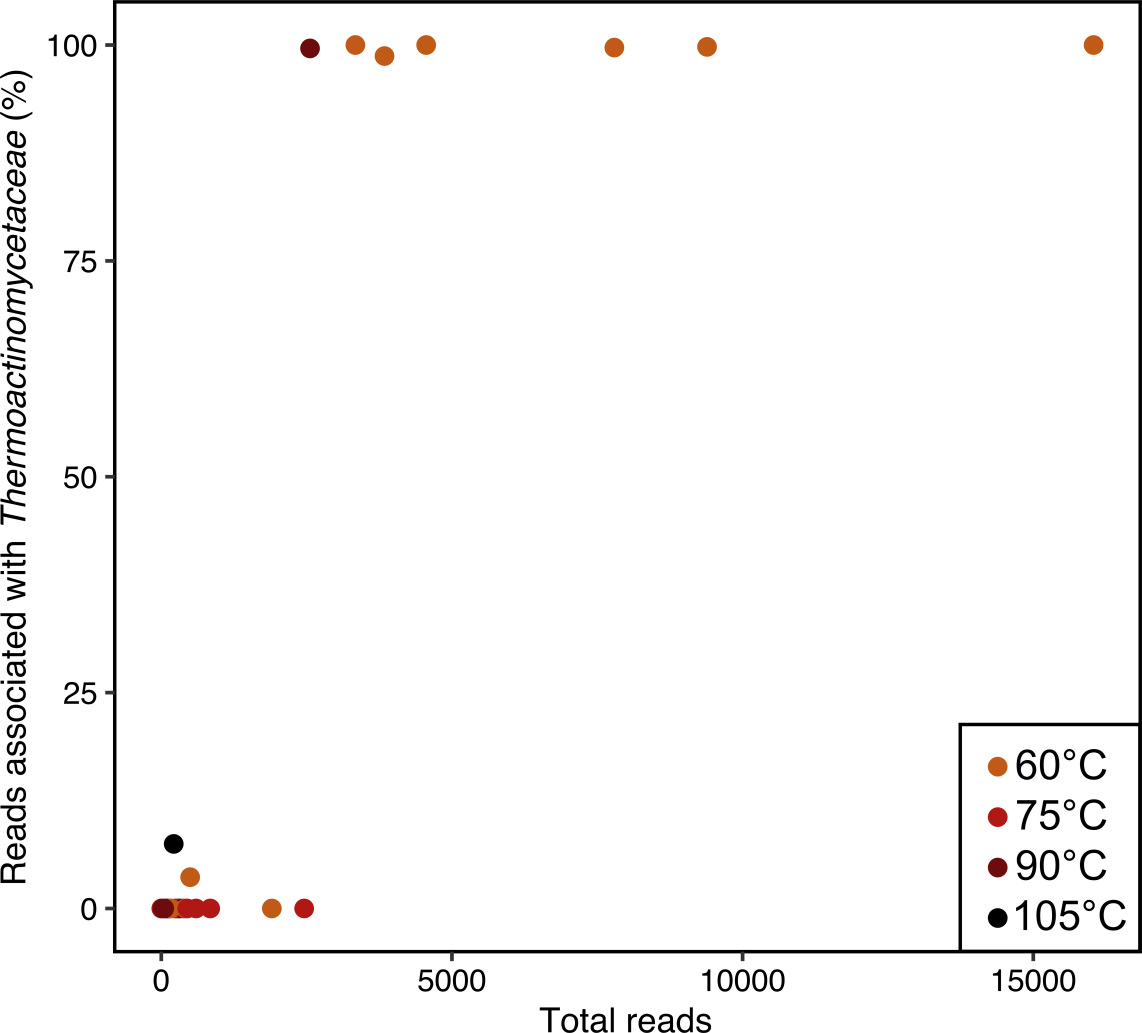
Scatterplot showing the total number of reads and the relative abundance of reads associated with family Thermoactinomycetaceae in all hydrated clay samples incubated at 60, 75, 90, or 105°C.

For bentonite hydrated and incubated at 60°C, all replicates of MX6 and MX7 had higher abundances of sequence reads than the remaining three bentonite samples (Fig. 4). The profiles of these samples were dominated (99–100%) by sequences associated with family Thermoactinomycetaceae (Fig. 4) and grouped together, separately from all other samples, in ordination space (Fig. 5). Overall, temperature was significantly correlated (*P* = 1 × 10^−4^) to both weighted UniFrac (Fig. 5A) and Bray–Curtis (Fig. 5B)-based ordinations (*R*^2^ = 0.54 and *R*^2^ = 0.58, respectively). The family Thermoactinomycetaceae was also present, though less abundant, in MX6 and MX7 incubated at 45°C (Fig. 4), but not at 75, 90, or 105°C, except in one replicate of GFM incubated at 90 and at 105°C (Fig. 4). Sequences associated with Thermoactinomycetaceae were only detected in one as-received clay sample (one replicate of Asha) at 1.5% relative abundance. The 16S rRNA gene profiles of all samples incubated at 75, 90, or 105°C had very little overlap, likely due to the very low sequencing depth for most of these samples (Fig. 4). In general, bentonite incubated at temperatures of 60°C or higher either had very few (average 241) sequencing reads, perhaps indicative of no or little microbial growth in the bentonite microcosm, or had relatively high sequencing depth and profiles dominated by sequences associated with family Thermoactinomycetaceae (Fig. 4 and 6).

Sequences associated with known SRB (*Desulfosporosinus* and Desulfovibrionaceae) were rarely detected in hydrated clay profiles, and only in samples with very low read counts, perhaps due to contamination of these samples from SRB culture samples with high abundances of these sequences. *Desulfosporosinus* sequences were detected in one MX9 replicate incubated at 75°C, which had a total of two reads, and Desulfovibrionaceae sequences were detected in an MX6 replicate incubated at 105°C that had 91 reads and in an Asha replicate incubated at 30°C that had 158 reads.

**Fig 7:**
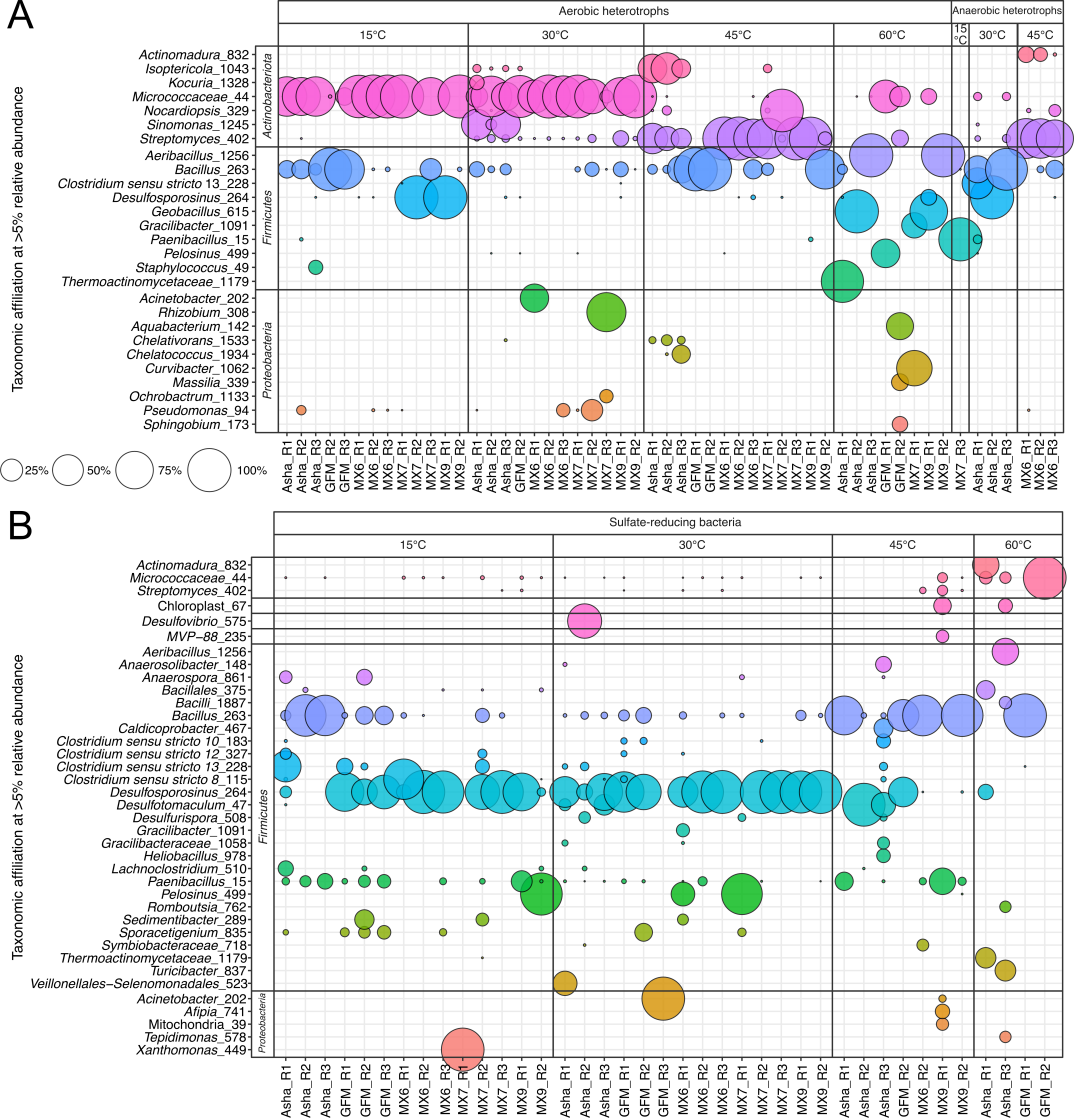
Bubble plots showing 16S rRNA gene profiles of heterotroph (**A**) and sulfate-reducing bacteria (**B**) cultures from hydrated bentonite microcosms incubated at 15, 30, 45, or 60°C. Only genera with a minimum relative abundance of 5% in at least one sample are displayed.

The heterotroph cultures inoculated with hydrated bentonite and incubated at 15, 30, or 45°C had 16S rRNA gene profiles dominated by sequences associated with similar taxa to those that dominated the hydrated clay profiles (Fig. 4 and 7A). For most aerobic heterotroph cultures incubated at 15 or 30°C, 16S rRNA gene profiles were dominated by sequences associated with Micrococcaceae. Exceptions to this were GFM replicates incubated at 15°C, which were dominated by sequences associated with *Bacillus*, and Asha replicates incubated at 30°C, which had a slightly higher relative abundance of *Sinomonas* (Fig. 7A). When grown under anoxic conditions at 30°C, profiles were instead dominated by sequences associated with *Bacillus* (Fig. 7A). With or without oxygen, heterotroph cultures incubated at 45°C were dominated by sequences associated with *Streptomyces* (Fig. 7A). There were three heterotroph samples with very high relative abundances of sequences associated with *Desulfosporosinus* (Fig. 7A). These samples each had a maximum of 16 sequence reads total, and thus, this ASV likely represents contamination from a concentrated SRB culture sample. The dominant taxon from high-temperature hydrated clay profiles, Thermoactinomycetota, was only detected in one aerobic heterotroph sample (Asha-R1 incubated at 60°C), where it comprised 95% of the 37,889 sequencing reads (Fig. 7A).

Like SRB cultures inoculated with as-received clay, the dominant genus of SRB in SRB cultures inoculated with hydrated clay was *Desulfosporosinus,* except for replicates of Asha bentonite incubated at 30 or 45°C, which were instead dominated by *Desulfotomaculum* (Fig. 7B). In SRB cultures incubated at 60°C, sequences associated with known SRB were only detected in one Asha replicate. Other SRB cultures at this temperature had poorly overlapping 16S rRNA gene profiles with dominant taxa including *Actinomadura*, Microccocaceae, *Aeribacillus*, and *Bacillus* (Fig. 7B).

## DISCUSSION

### Cultivation of heterotrophs from as-received bentonite

The cultivation results demonstrated that microorganisms are culturable from a variety of as-received clays at a temperature of 30°C as has previously been shown (18), but also at temperatures of 15, 45, and 60°C (Fig. 2). The abundance of culturable microorganisms from as-received clay was similar regardless of incubation temperature. That said, at the highest tested temperature of 60°C, aerobic heterotrophs were not culturable from two bentonite samples, and anaerobic heterotrophs were only culturable from one bentonite sample, suggesting that some as-received bentonite samples may have microorganisms better adapted to withstanding high temperature than others (Fig. 2). Alternatively, this result could reflect the proliferation or survival of culturable thermophiles in some bentonite samples (i.e., those where they are detectable via cultivation) and non-culturable thermophiles in other bentonite samples (i.e., those where culturable microorganisms were not detected).

### Cultivation of heterotrophs from hydrated bentonite

Previous experiments showed that there is often an increase in abundance of culturable heterotrophs when water is added to bentonite, provided that swelling pressure is sufficiently low, either from incubating hydrated bentonite in an unconfined space or from incomplete saturation of a system that will eventually experience elevated swelling pressure (9, 21, 22). Similar to these previous experiments conducted at 30°C, the present study also showed an increase in the abundance of culturable aerobic heterotrophs after bentonite hydration and incubation at 30°C, and also often at 15 and 45°C, but never at 60°C (Fig. 2). Similar increases were generally not observed for anaerobic heterotrophs, but this study was conducted under oxic conditions, which even in the absence of elevated temperature would be expected to limit or preclude growth of anaerobic heterotrophs. The decreased number of hydrated clays with culturable anaerobic heterotrophs detected, compared to as-received clays, could possibly be a result of aerobic heterotrophs outcompeting anaerobic heterotrophs because of the oxic incubation conditions (Fig. 2). Although the experiments presented here were conducted using uncompacted bentonite, these results agree with a previous study that showed that the abundance of culturable microorganisms in highly compacted bentonite (i.e., 1.7 g/cm^3^) incubated at 60°C did not increase (8). In a separate study of Bavarian clay slurries, no microbial activity was measured in 60°C incubations (13). However, microorganisms remained viable throughout incubation at elevated temperature and were still culturable after incubation (8, 13). Similar results, showing no increase in abundance but residual low numbers of culturable microorganisms after high-temperature incubation, were also reported for studies on BCV bentonite (14–16). Combined with the results of the current study, cultivation results suggest that the microbial communities within bentonite clay may prefer more moderate temperatures (i.e., 15–45°C) but that some members of the community are able to survive incubation at elevated temperatures. However, abundances of culturable aerobic heterotrophs incubated at 60°C, compared to lower temperature incubations, do not necessarily preclude the possibility of thermophilic growth in these hydrated bentonite microcosms, but instead could be a reflection of unsuitable cultivation conditions.

### Cultivation and DNA analysis of sulfate-reducing bacteria

Consistent detection of SRB at low abundance, and cultivation of SRB from some clays at all tested incubation temperatures, suggests the presence of bentonite-associated thermotolerant or thermophilic SRB (Fig. 2). Similar to cultivation results, for both as-received and hydrated bentonite, sequences associated with known SRB were rarely detected and never at high relative abundances (Fig. 3 and 4). The SRB cultures inoculated with both as-received and hydrated clay were typically dominated by sequences associated with *Desulfosporosinus*, which is a commonly detected genus of SRB in bentonite (5, 6, 18, 21, 22). Coupled with consistently low abundances of culturable SRB from both as-received and hydrated clay, the results indicate that no growth of SRB in bentonite occurred under any tested conditions. However, SRB are known to be obligate anaerobes, and the bentonite microcosms presented here were incubated under oxic conditions. Future bentonite studies conducted under anoxic conditions will be important for confirming a lack of thermophilic SRB growth in bentonite.

### Mesophilic bentonite microbial communities

A DNA-based analysis was included to examine shifts in bentonite community composition before and after hydration and incubation at elevated temperature as a second tool to identify microbial growth at elevated temperature. In agreement with a previous experiment conducted at 30°C using similar conditions, ASVs associated with *Streptomyces* dominated the microbial profiles of hydrated bentonite incubated at 30°C, as well as 15 and 45°C (22). At 15 and 30°C, sequences associated with Micrococcaceae were also relatively abundant, as were sequences associated with *Nocardiopsis* in bentonite incubated at 30 or 45°C. *Streptomyces*, Micrococcaceae, and *Nocardiopsis* are all members of the phylum Actinobacteriota and are aerobic heterotrophs best known for their production of biotechnologically and medically relevant secondary metabolites (23–25), with some members of family Micrococcaceae being cold-adapted (26). The fact that these taxa increased in relative abundance after bentonite hydration and incubation, compared to the starting material, implies growth within the clay. This is further supported by 16S rRNA gene profiles of hydrated clay culture biomass that were dominated by sequences associated with these same taxa, as well as an increase in culturable aerobic heterotrophs observed for these samples after hydration.

### Thermophilic bentonite microbial communities

When sequencing depth was sufficiently high for bentonite incubated at temperatures of 60°C or higher, 16S rRNA gene profiles were dominated by sequences associated with the family Thermoactinomycetaceae (Fig. 4 and 6). This was most apparent in the hydrated MX6 and MX7 replicates incubated at 60°C, where sequences associated with this family made up 99-100% of the sequencing reads (Fig. 4 and 6). Family Thermoactinomycetaceae consists of thermophilic aerobic heterotrophs, often capable of producing endospores (27). Sequences associated with this family were not detected in as-received MX6 or MX7 replicates. The comparative high relative abundance of sequences associated with this family in hydrated clays with high sequence read counts suggests that microorganisms belonging to family Thermoactinomycetaceae grew in several high-temperature microcosms (e.g., MX6 and MX7, 60°C). A similar observation was reported in a study of Bavarian bentonite in a slurry with Opalinus clay porewater, where no microbial activity was measured in 60°C microcosms, but DNA sequencing revealed a shift in microbial community composition from starting material to after hydration and incubation. In this study, after incubation, microbial profiles were dominated by the thermophilic species *Caldinitratiruptor microaerophilus* (13), which is a facultatively microaerophilic, heterotrophic, nitrate-reducing, non-spore-forming bacterium with an optimal growth temperature of 65°C (28). Together, these results suggest that viable thermophilic microorganisms were present in several bentonite samples, potentially surviving as spores, and can proliferate when provided with suitable conditions (e.g., water and an elevated growth temperature). If other conditions (e.g., pressure, degree of saturation, oxygen presence) permit, thermophiles, such as members of family Thermoactinomycetaceae, could be expected to grow within or surrounding a DGR. Methodologically, these results indicate that cultivation may not be a reliable method for demonstrating decreased abundance of viable microorganisms at elevated temperature. Based on the DNA sequence results of the present study, the cultivation-based abundance results are likely an underestimate of the abundance of viable thermophiles in bentonite. Sequences associated with Thermoactinomycetaceae were rarely detected in cultures from hydrated clays, further suggesting that the cultivation conditions used here, and commonly elsewhere, were not ideal for these microorganisms.

### Conclusion

Temperature is expected to vary substantially within the timeframe of a DGR. Although temperature-related design specifications have been established to ensure the stability of copper and bentonite at elevated temperature, the impact of temperature on microbial communities in bentonite has rarely been studied. The results of this study demonstrate that temperature does influence which microorganisms grow in hydrated bentonite and that thermophilic microorganisms may exist in as-received bentonite at very low abundance. At moderate incubation temperatures (i.e., 15–45°C), several taxa that were detectable in as-received bentonite proliferated and were confirmed to be viable because the same taxa were detected in 16S rRNA gene profiles of culture biomass from hydrated clay cultures. At high incubation temperatures (i.e., 60–105°C), DNA evidence suggests that thermophilic taxa grew that were not detected in as-received clay, despite no growth observed in the cultures. These results indicate that as-received bentonite contains numerous microbial cells at low relative abundance that can proliferate under distinct incubation temperatures, but may not be detectable using a direct DNA-sequencing from as-received bentonite approach. These results further suggest that cultivation may underestimate the abundance of thermophilic microorganisms in bentonite. This highlights the importance of a multifaceted approach to studying community composition of low biomass bentonite clays. Future studies should continue to incorporate different temperatures into their experimental design to test the effect of temperature combined with other parameters that are expected to vary within a DGR spatially and temporally (e.g., swelling pressure, water activity, and oxygen) and others that are inherent to the selected host rock (e.g., groundwater composition). Future experiments should also consider longer incubation times, which may allow for the growth of slow-growing thermophiles. This will allow for a more detailed understanding of the specific microorganisms that may proliferate in a DGR when swelling pressure is sufficiently low (e.g., during periods of incomplete saturation) to not suppress microbial growth altogether.

## MATERIALS AND METHODS

### Bentonite background

Five different manufactured sodium-type bentonite clays were used in this study and are referred to throughout as Asha, MX6, MX7, MX9, and GFM. Asha is a bentonite sourced from Kutch, India, and manufactured by Ashapura Minechem Co. This as-received clay was previously characterized using DNA sequencing and cultivation (18). Both MX6 and MX7 are Wyoming bentonite and were used in borehole modules in the Grimsel Underground Research Laboratory (5, 6). Clay MX9 is a Wyoming MX-80 bentonite that was characterized in the same study as Asha bentonite (18) and separately in a hydrated clay microcosm study (22). Gapfill material (GFM) was made from Wyoming MX-80 bentonite through a roll compaction process. It was included in two studies that aimed to identify the minimum dry density necessary to suppress microbial growth using pressure vessels (21). The mineral composition of each as-received bentonite sample used to fill pressure vessels was determined using quantitative XRD analysis, performed by Activation Laboratories using a Bruker D8 Endeavour diffractometer equipped with a Cu X-ray source. For clay speciation, a portion of each sample was dispersed in distilled water, and the <25 µm size fraction was separated by gravity settling. The proportion of clay minerals was calculated using the ratios of their basal peak areas. The PDF-4/Minerals database (International Centre for Diffraction Data) was used for mineral quantification, and the “Rietveld method” (29) was used to determine the quantity of each crystalline mineral.

### Microcosm experiment setup

From each as-received clay, cultivation and DNA analysis were conducted with no hydration nor prior incubation of clay at elevated temperature (Fig. 1A). Separately, each as-received clay was hydrated and incubated at various temperatures (Fig. 1B). Sample names, including “R1,” “R2,” and “R3,” refer to biological replicates.

For hydrated clay microcosms, as-received bentonite was mixed with a predetermined (via trial and error) volume of autoclaved type I water to achieve a water activity of 0.99 (Table 2), which resulted in a wet clay, but not a slurry. The hydrated clay was mixed by hand with sterile nitrile gloves until a homogenous mixture was achieved. A WP4 Dew Point Potentiometer (Meter Group, USA) was used to ensure that the water activity of hydrated bentonite was 0.99. For 15–60°C microcosms, three 80 g aliquots of bentonite were mixed with water, and each was then split into 4 × 20 g aliquots for each of the four temperatures of a given replicate. As 75–105°C microcosms did not include a cultivation analysis, a smaller mass of 10 g of bentonite was used for each of these microcosms.

**TABLE 2:** Volumes of type I water added to 10 or 80 g of each type of bentonite to achieve a water activity of 0.99

Bentonite	Volume of water (mL) to add to 10 g of bentonite	Volume of water (mL) to add to 80 g of bentonite
Asha	3.5	28.0
MX6	4.3	34.0
MX7	4.4	35.0
MX9	3.0	24.0
GFM	4.5	36.0

For each type of bentonite, a total of 21 microcosms were set up in sterile 250 mL mason jars. Triplicate jars were incubated at 15, 30, 45, 60, 75, 90, or 105°C for one week (Fig. 1). Setup was performed on the bench next to a Bunsen burner to avoid contamination of microcosms, after which hydrated clay microcosms were incubated under oxic conditions for one week. The one-week incubation time was selected because a previous hydrated clay microcosm study using a similar experimental setup (other than temperature) observed increases in the abundance of culturable heterotrophs in several microcosms within the first week of incubation, after which abundances remained relatively consistent (22). After incubation, the jars were shaken to mix the clay, and a subsample was collected for cultivation and for DNA extraction. Cultures were set up immediately, and bentonite intended for DNA extraction was stored at −20°C until DNA extraction.

### Cultivation from microcosms

For each bentonite sample (as-received or hydrated), 2 g of bentonite was added to 18 mL of phosphate buffered saline solution to create a 1:10 dilution (PBS; 8 g NaCl, 0.2 g KCl, 1.44 g Na_2_HPO_4_, and 0.24 g KH_2_PO_4_ in a final volume of 1 L type I water, with pH adjusted to 7.4 using HCl) in a 25 mL tube. Tubes were vortexed horizontally at low speed for 30 minutes to disperse the bentonite in the PBS. After vortexing, serial 1:100 and 1:1000 dilutions were made in PBS.

Three types of cultures were set up: aerobic heterotrophs, anaerobic heterotrophs, and SRB. For heterotroph cultures, 100 µL of dilution (1:10 and 1:100 for anaerobes, and all three dilutions for aerobes) was added to triplicate Reasoner’s 2A (R2A) agar (M1687; HiMedia Laboratories) plates and spread using sterile glass beads. An agar concentration of 1.5% was used for plates incubated at 15 and 30°C, while a concentration of 3% was used for plates incubated at 45 or 60°C, as 1.5% agar plates were partially melted during incubation at elevated temperatures. For SRB, 15 test tubes per sample filled with 9 mL of SRB medium (M803; HiMedia Laboratories) were inoculated with 1 mL of dilution (five test tubes per dilution).

All aerobic heterotroph cultures were incubated under oxic conditions for five days, and anaerobic heterotroph and SRB cultures were incubated under anoxic conditions for 28 days. For cultures incubated anoxically, tubes or plates were placed in airtight bags (Uline; ~30 tubes or 24 plates per bag). To establish anoxic conditions, a GasPak EZ anaerobe sachet (BD) was added to each bag, which was then flushed with a 90% N_2_ and 10% CO_2_ gas mixture for two to three minutes. Each bag also included an anaerobic indicator strip (BD) to verify anoxic conditions throughout incubation.

For each as-received (un-hydrated) clay, a set of cultures was incubated at each of 15, 30, 45, and 60°C (Fig. 1). For hydrated clays, cultures were incubated at the same temperature as the hydrated bentonite used to inoculate the culture (Fig. 1). Overall, for each of the five clay samples, triplicate sets of cultures, each derived from a biological replicate mason jar, were incubated at 15, 30, 45, and 60°C. Although hydrated clay microcosms were also incubated at 75, 90, and 105°C, cultures were not incubated at these temperatures because agar would melt.

After incubation, colonies on R2A agar plates were counted, and these numbers were used to calculate the values of CFU/g. For SRB cultures, tubes were examined for SRB activity, which was identifiable by the presence of a black ferrous sulfide precipitate produced by a reaction between SRB-derived sulfide and the ferric ammonium sulfate present in the SRB medium. The number of cultures positive for SRB activity for each dilution was used to calculate the most probable number (MPN) of SRB. Biomass was collected from each culture where growth was evident. For heterotroph cultures, colonies were collected on sterile foam-tipped applicators (Puritan), and for SRB cultures, 1 mL of each positive culture was collected and centrifuged at 8000 × *g* for 10 minutes to pellet cells. For a given bentonite sample, biomass from all positive 1:10 dilution SRB cultures was collected together, and biomass from all positive 1:100 and 1:1000 dilution SRB cultures was collected separately. Biomass was stored at −20°C until DNA extraction.

Because there was a visually apparent effect of temperature and hydration on aerobic heterotroph culture counts, an ANOVA was used to statistically test the effect of temperature, with or without hydration, on aerobic heterotroph culture counts for each of the five bentonite samples. Each sample group consisted of the three replicate samples for a given bentonite, hydration condition (i.e., hydrated or dry), and temperature. As the ANOVA resulted in a significant *P* value (<0.05), a Tukey’s Honestly Significant Difference (HSD) test was used to determine which sample conditions resulted in significantly different aerobic heterotroph counts.

### DNA extraction from bentonite and culture biomass

Genomic DNA was extracted from 50 mg of the 105 (5 clay types × 7 temperatures × triplicate) hydrated clay samples and from duplicate aliquots of each of the five as-received clay samples using the DNeasy PowerSoil Pro Kit (Qiagen). Genomic DNA was extracted from culture biomass using the DNeasy UltraClean Kit (Qiagen). The manufacturer’s instructions were followed with the following exceptions. After the addition of lysis solution, PowerBead tubes were incubated at 70°C for 10 minutes, followed by bead beating for 45 seconds using a FastPrep-24 Classic Instrument (MP Biomedicals) set to 5.5 m/s. DNA was eluted in 60 µL for PowerSoil extractions and in 50 µL for UltraClean extractions. Genomic DNA concentration was measured using the Qubit dsDNA HS Assay Kit (Invitrogen). After extraction, DNA samples were stored at −20°C until further analysis.

### Amplicon sequencing of bentonite and culture biomass

Despite undetectable DNA concentrations for bentonite samples, the V4-V5 region of the 16S rRNA gene was amplified in triplicate using universal primers 515F-Y (30) and 926R (31), modified to include a six-base index sequence, Illumina flow cell binding sites, and sequencing primer binding sites (32), for each genomic DNA sample (hydrated clay, as-received clay, culture biomass). All PCR mixtures were prepared in a PCR hood (AirClean Systems) with ISO 5-HEPA-filtered air, and all surfaces were wiped with 70% ethanol and UV-treated for 15 minutes before each use. Tubes, water, and BSA used for PCR were also UV-treated on a UV transilluminator (ProteinSimple) using 302 nm wavelength light. Each PCR consisted of 1 × ThermoPol Buffer (New England BioLabs), 0.2 µM forward and reverse primer, 200 µM dNTPs, 15 µg BSA, 0.625 U hot start Taq DNA polymerase (New England BioLabs), and 1 µL of template DNA in a final volume of 25 µL. The following thermal cycle conditions were used: initial denaturation at 95°C for five minutes, followed by 35 (cultures) or 40 (clay) cycles of 95°C denaturation for 30 seconds, 50°C annealing for 30 seconds, and 68°C extension for seven minutes, followed by a final extension step for seven minutes at 68°C. No template controls (NTCs) were included in each 96-well plate to test for contamination between wells, and additional NTC and positive controls were included in tubes to test for master mix contamination and appropriate amplification, respectively.

Triplicate PCR products were imaged on a 1% agarose gel stained with ethidium bromide, and then triplicates were pooled and quantified on a second 1% agarose gel stained with GelRed (Biotium). Based on gel quantification, equimolar quantities of each sample were pooled. Kit controls and NTCs were added to the pool, regardless of visible amplification, at a volume of 5 µL. This pool was electrophoresed on a 1.5% agarose gel stained with ethidium bromide, and the band corresponding to the amplicons was excised and purified using the Wizard SV Gel and PCR Clean-Up System (Promega). The purified pool was quantified using the Qubit dsDNA High Sensitivity Assay Kit (Invitrogen) and with qPCR using P5 and P7 primers (Illumina). The library was denatured and diluted following Illumina guidelines (Document No. 15039740 v01) and then spiked with PhiX Sequencing Control v3 (Illumina).

All as-received clay and associated cultures were sequenced on a MiSeq run with a cluster density of 659,000 clusters/mm^2^, an average Q30 of 93.0%, and 92.5% of clusters passing filter. All hydrated clay samples were sequenced on the second MiSeq run with a cluster density of 774,000 clusters/mm^2^, an average Q30 of 90.0%, and 91.4% of clusters passing filter. All culture samples from hydrated clay were sequenced on the third MiSeq run with a cluster density of 813,000 clusters/mm^2^, an average Q30 of 89.5%, and 93.2% of clusters passing filter.

### Microcosm sample sequence analysis

Sequences were demultiplexed using Local Run Manager version 4. Demultiplexed reads were further analyzed using Quantitative Insights Into Microbial Ecology 2 (QIIME 2) (33) including Divisive Amplicon Denoising Algorithm 2 (DADA2) (34). Paired-end reads were imported into QIIME 2, and DADA2 was used to remove low-quality reads and primer sequences and to truncate reads to 250 bases. Next, sequences were denoised and dereplicated, and amplicon sequence variants (ASVs) were generated, again using DADA2 through QIIME 2. Taxonomy was assigned to ASVs using a naïve Bayes classifier pre-trained with SILVA database release 138. The Decontam R package (35) was used to identify potential contaminant sequences. A threshold of 0.5 was selected by examining histograms of Decontam score distribution. The ASV table from each sequencing run was processed with Decontam separately, and the ASV table, including both dry clays and associated cultures, was further split by sample type (i.e., clay and culture) prior to processing. For clay samples, several ASVs flagged as contaminants were associated with thousands of reads in culture samples and only a few reads in one or a few controls. As such, any ASV flagged as a contaminant that was associated with >1,000 reads in at least one culture and <50 reads in any control was removed from the list of contaminants. Contaminant ASVs were removed from the ASV tables prior to subsequent analysis. After Decontam, ASV tables were merged using qiime feature-table merge. For the purpose of generating bubble plots, the merged table was collapsed to the genus level using qiime taxa collapse.

A version of the ASV table, including only bentonite samples, was normalized to 1,000 reads using scaling with ranked subsampling (SRS) through the SRS package in R (36). This normalized table was used to calculate Bray–Curtis and weighted UniFrac distances using the R packages Vegan and GUniFrac, respectively. Vegan was additionally used to add vectors and taxa bubbles to create triplots (presented in Fig. 4): parameters were represented by vectors if they were significantly (*P* ≤ 0.05) correlated to the ordination with a minimum *R*^2^ value of 0.2, and taxa with minimum relative abundances of 5% across the data set were represented as bubbles.

## Data Availability

All sequence data were deposited in the European Nucleotide Archive with accession number PRJEB79490. A merged ASV table including all samples and controls prior to Decontam processing, a merged ASV table including all samples after Decontam processing (used for all analysis other than generating bubble plots), and a genus-collapsed version of the ASV table after Decontam processing (used to generate bubble plots) are available as Table S1. The genus_number column of the genus-collapsed table corresponds to the genus numbers in each bubble plot.

## SUPPLEMENTAL MATERIAL

Figure S1 msphere.00313-25-s0001.docx Bubble plot showing the 16S rRNA gene profiles of kit controls (KC) from PowerSoil and UltraClean DNA extraction kits, as well as PCR no-template controls (NTC).

Table S1 msphere.00313-25-s0002.xlsx Amplicon sequence variant (ASV) tables of all samples before/after processing with Decontam and after genus collapsing.

ASM does not own the copyrights to Supplemental Material that may be linked to, or accessed through, an article. The authors have granted ASM a non-exclusive, world-wide license to publish the Supplemental Material files. Please contact the corresponding author directly for reuse.
