# Supplementary material for: Effect of temperature on microbial communities in bentonite for use in engineered barrier systems
Source: mSphere. 2025 Aug 8;10(9):e00313-25. doi: 10.1128/msphere.00313-25 (PMC12482174; doi:10.1128/msphere.00313-25)
Supplement: Figure S1 — Bubble plot showing the 16S rRNA gene profiles of kit controls (KC) from PowerSoil and UltraClean DNA extraction kits, as well as PCR no-template controls (NTC). [file msphere.00313-25-s0001.docx]

## **Effect of temperature on microbial communities in bentonite for use in engineered barrier systems**

## Rachel C. Beaver^1^, Cailyn M. Perry^1^, Chang Seok Kim^2^, Josh D. Neufeld^1^*

^1^Department of Biology, University of Waterloo, Waterloo, Ontario, Canada

^2^Nuclear Waste Management Organization (NWMO), Toronto, Ontario, Canada

Running head: Temperature and bentonite microbial communities

*Corresponding author

Department of Biology, University of Waterloo, 200 University Avenue West, Waterloo, Ontario, N2L 3G1, Canada. Tel. +1 519-888-4567; Fax +1 519-746-0614

E-mail: jneufeld@uwaterloo.ca

**SUPPLEMENTAL MATERIAL**

Figure S1. Bubble plot showing the 16S rRNA gene profiles of kit controls (KC) from PowerSoil and UltraClean DNA extraction kits, as well as PCR no template controls (NTC). Only ASVs with a minimum relative abundance of 5% in at least one sample are included. The bar plot on top shows the total number of sequencing reads per sample.

**Supplemental data files**

Supplemental_file_1.xlsx includes amplicon sequence variant (ASV) tables of all samples before/after processing with Decontam, and after genus collapsing.
